# Health Inequity in the Distribution of Diseases Among Adults in the City of Pécs, Hungary, 2024

**DOI:** 10.3390/healthcare13060681

**Published:** 2025-03-20

**Authors:** Addisu Alemayehu Gube, Szimonetta Lohner, Jozsef Vitrai

**Affiliations:** 1 Doctoral School of Health Sciences, Faculty of Health Sciences, University of Pécs, 7622 Pécs, Hungary; vitrai.jozsef@gmail.com; 2 Department of Public Health Medicine, Medical School, University of Pécs, 7622 Pécs, Hungary; 3 Cochrane Hungary, Medical School, University of Pécs, 7622 Pécs, Hungary

**Keywords:** health inequity, neoplasms, circulatory diseases, Pécs, Hungary

## Abstract

**Background:** Health inequalities between citizens of different European countries and between socially advantaged and disadvantaged groups are seen as challenges to the EU’s commitment to solidarity, social and economic cohesion, human rights, and equality of opportunity. This study aimed to assess inequity in the distribution of specific communicable and non-communicable diseases among the adult population of Pécs City, Hungary. **Methods:** This is an ecological study combined with health inequity analysis. The study population comprised adult patients living in the city of Pécs who were treated for circulatory, neoplasm, or respiratory diseases in 2019. Hospitalization and local socioeconomic data by zip codes were obtained from the National Health Insurance Fund of Hungary and the Hungarian Central Statistical Office. Diseases were classified according to the International Classification of Diseases, 10th revision (ICD-10). The differences and ratios of the proportions of treated diseases were calculated, along with the concentration index (C). Zip codes were ranked into categories 1 to 5 based on socio-demographic variables: education, employment status, and apartment ownership. A *p*-value of less than 0.05 was used as the cut-off point for statistical significance. IBM SPSS version 25 and STATA version 14.0 software were used for data analysis. **Results:** All 36 zip codes in the city of Pécs were included in this study. Analysis based on employment status showed a higher prevalence of neoplasms in areas with higher unemployment (C = −0.0528; 95% CI = −0.0975, −0.0080). In terms of apartment ownership, neoplasms (C = −0.0742; 95% CI = −0.1102, −0.0382) and circulatory diseases (C = −0.0280; 95% CI = −0.0520, −0.0039) were more prevalent in zip codes with fewer apartment owners. **Conclusions:** This study identified significant inequity in the distribution of neoplasms and circulatory diseases among the adult population of Pécs, especially in areas where the low socioeconomic segment of the population lives. Efforts should be made to establish tailored interventions such as targeted healthcare funding and employment programs for the unemployed and National Housing Trust Fund for those who do not own houses through multisectoral collaboration among healthcare providers, employers, governors, and policymakers.

## 1. Introduction

Health inequity, a form of health inequality, denotes unjust, preventable, and unnecessary differences in health outcomes. In this sense, health inequities are systematic differences in health that could be avoided by reasonable means [1,2]. These inequities can be horizontal, when people with the same needs do not have access to the same resources, or vertical, when people with greater needs are not provided with greater resources [3].

Health equity is a fundamental element of the United Nations Sustainable Development Goals (SDGs), particularly SDG 3, which aims to “Ensure healthy lives and promote well-being for all at all ages”, and SDG 10, which highlights the importance of reducing “inequality within and among countries”. Achieving health equity is key for achieving many other SDGs [4].

Health inequities are measured using simple measures (e.g., differences and ratios) or complex measures (e.g., the slope index of inequality and concentration index) [5,6].

Health inequalities reduce economic and social productivity and lead to higher healthcare and welfare costs. In the European Union, inequalities in health are estimated to cost EUR 980 billion per year, or 9.4 percent of the European GDP [7]. These health inequalities challenge the EU’s values of solidarity, social and economic cohesion, human rights, and equality of opportunity [8].

Health outcomes and the presence of health inequities are influenced by a range of social, economic, and environmental determinants of health, i.e., conditions in which people are born, grow, live, work, and age [9]. The theory of fundamental causes of health inequalities posits that socioeconomic status (SES) influences a wide range of health conditions. There is a clear correlation between SES and various factors that elevate the risk of disease and mortality. The distribution of resources plays a critical role in shaping the relationship between SES and health outcomes or mortality. This connection persists over time, even as the specific mechanisms linking SES to health outcomes are replaced by new ones [10].

The WHO Health Equity Status Report indicates that 90% of health inequalities can be explained by factors such as financial insecurity, poor quality housing and neighborhood environment, social exclusion, lack of decent employment opportunities, and poor working conditions [11].

Health outcomes worsen as SES decreases, a global trend observed across income levels [12]. People in low socioeconomic classes are more likely to have high levels of unemployment, live in overcrowded environments, have a higher proportion of single-parent families, as well as poorer health, higher rates of hospitalization, lower life expectancy, and premature mortality for all causes [13].

Health inequalities persist across Europe, both within and between countries, influenced by factors like education, economy, housing, environment, and healthcare. Lower SES is linked to poorer health, higher disease rates, and reduced life expectancy [9].

Inequalities in life expectancy by education are greater among men, especially in Central and Eastern Europe. In Slovakia, Poland, and Hungary, 30-year-old men with low education live over 10 years less than those with a high education [14].

In Europe, there are substantial disparities in the prevalence of chronic diseases by income group. On average across EU countries, 27% of people aged 65 and over in the highest income quintile reported at least two chronic diseases, compared with 46% for those in the lowest income quintile [15]. Similar patterns can be seen for activity limitations, as well as for unmet medical and dental care needs [14].

In Hungary, significant health inequities persist, influenced by geography and education. Men in Central Hungary live nearly 7 years longer than those in Northern Hungary, while women have an 8 year gap. Men with only primary education live 12 years less than those with higher education, with a 5.6 year gap for women. Treatment waiting times vary widely, from 31 days in Central Hungary to 110 days in Southern Transdanubia. Life expectancy at age 45 also varies by settlement income, with gaps of 4.6 years for women and 6.9 years for men between the highest and lowest income groups [16,17].

The aim of the present study was to identify the health inequity in the distribution of specific communicable and non-communicable diseases among adults living in the Hungarian city, Pécs.

## 2. Materials and Methods

### 2.1. Study Design

The study design of this study is an ecological study combined with health inequity analysis.

### 2.2. Setting

Pécs is a city in south-western Hungary, the fifth largest settlement in the country. It is the administrative center of Baranya County and lies near the Croatian border, about 45–50 km west of the river Danube. The resident population of Pécs on 1 October 2022 was 139,330 people, which accounted for 40.4% of the total population of Baranya County. In 2019, the average life expectancy at birth was 73.02 years for men and 79.53 years for women, which is higher than the national average [18].

### 2.3. Participants

The study population consisted of all adults aged 18 years or older living in Pécs in 2019 and treated for circulatory, cancer/neoplasms, or respiratory diseases, regardless of whether the treatment occurred in hospital or outpatient clinics within or outside of Pécs.

### 2.4. Variables

The dependent variable is health inequity. The independent variables include socio-demographic variables such as education, employment, apartment ownership, and zip codes of the Pécs city. The disease variables are circulatory diseases (ICD-10, I00–I99), neoplasms (ICD-10, C00–C97), and respiratory system diseases (ICD-10, J00–J99).

#### Operational Definitions

**Circulatory diseases**: defined for the purpose of this study based on International Classification of Diseases version 10 (ICD-10), which is designated a code of I00–I99 [19].

**Neoplasms**: defined for the purpose of this study based on International Classification of Diseases version 10 (ICD-10), which is designated a code of C00–C97 [19].

**Respiratory system diseases:** defined for the purpose of this study based on International Classification of Diseases version 10 (ICD-10), which is designated a code of J00–J99 [19].

### 2.5. Data Sources

Secondary data on the proportion of patients treated for the diseases were obtained from the National Health Insurance Fund of Hungary (NEAK), and data on socio-demographic variables such as education, employment, and apartment ownership by zip codes of the city of Pécs were obtained from the Hungarian Central Statistical Office (KSH).

### 2.6. Study Size

Since all data were included, no sampling procedure was necessary.

### 2.7. Statistical Methods

To measure health inequity, we used both simple and complex measures. Simple measures included absolute inequality (difference) and relative inequality (ratio), while the concentration index was used as a complex measure [20]. Simple measures make pairwise comparisons of health between only two subgroups, such as the most and least wealthy. Complex measurements, on the other hand, make use of data from all subgroups to assess inequality. It produces a single number that is an expression of the amount of inequality existing across all subgroups of a population.

The concentration index takes values between −1 and +1; with the value of 0 indicating the presence of equality. A negative value indicates that a ‘bad’ health outcome is higher among poorer populations [5].

We divided the city into different areas based on zip codes and ranked them based on socio-demographic variables from 1 to 5 quintiles. Where for education, category 1 indicates lowest proportion of educated residents, while 5 indicates the highest proportion. For employment, category 1 indicates lowest proportion of employed individuals, while 5 indicates highest proportion. For apartment ownership, 1 indicates lowest proportion, while 5 indicates highest proportion of owner-occupied apartment.

The software IBM SPSS Version 25 and STATA version 14.0 were used for analyzing the data. Both descriptive and health inequity analyses were carried out, with *p*-value less than 0.05 considered as statistically significant.

### 2.8. Ethical Considerations

To access the medical or health care data, legal permission was obtained from the National Health Insurance Fund (NEAK) of Hungary. For socioeconomic data, legal permission was obtained from the Hungarian central statistical office (KSH).

## 3. Results

### 3.1. Socio-Demographic Variables

We investigated the following three socio-demographic variables: education, employment status, and apartment ownership. There was a notable contrast in educational attainment among areas: in the area with the highest number of residents holding university or college degrees they were representing 30.66% of the population, while in the area with the lowest number of degree holders this percentage was only 7.2%. There was an area represented by a specific zip code where the number of those with educational status of grade 8 and below was especially high, with 46.85% of the population living in this area. Employment status ranged between 30.62% and 46.23%, while apartment ownership ranged between 61.79% and 96.47% (Table S1).

### 3.2. Descriptive Statistics

We collected information for all the 36 zip codes in Pécs. Neoplasms showed varying prevalence in the different areas represented by different zip codes, with the highest proportion of neoplasms being at 3.5% (76 patients out of 2174) in one of the areas and the lowest at 1.2% (11 patients out of 936) (Figure S1). Circulatory diseases were more common, reaching up to 41.7% (686 patients out of 1646 inhabitants), while the minimum prevalence was 27.3% (1831 patients out of 6714 inhabitants) (Figure S2). Respiratory system diseases had a maximum prevalence of 29.5% (2451 patients out of 8305 inhabitants) and a minimum prevalence of 16.1% (207 patients out of 1290 inhabitants) (Figure S3).

### 3.3. Inequity Analysis

#### 3.3.1. Simple Measures of Inequity

The table below indicates slight differences in the proportion of patients treated with respiratory diseases and neoplasms based on educational status of the population, whereas no difference detected in case of circulatory disease. Based on the employment status of the population, there is slight difference in the prevalence of respiratory diseases, while no difference found in the distribution of circulatory disease and neoplasms. For the three diseases, no difference in prevalence was found based on apartment ownership (Table 1).

#### 3.3.2. Complex Measures of Inequity

For complex measures of inequity, we used concentration index and curve.

##### Disease Proportion Based on Education

The table below summarizes the concentration index for the three diseases based on educational status of population, and none of the results were statistically significant. It indicates that the prevalence of the diseases is not associated with educational status of the population in the zip codes (Table 2).

##### Disease Proportion Based on Employment

**Figure 1:**
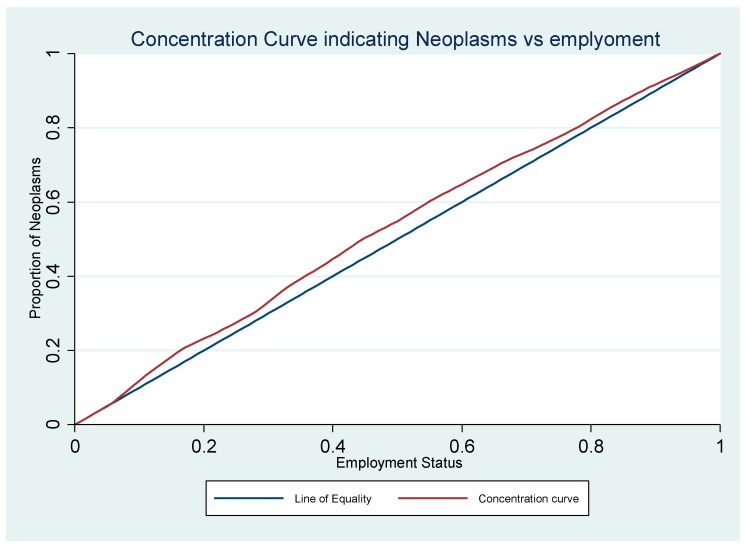
The concentration curve based on proportion of patients treated with neoplasms and the employment status in Pécs City.

In the table below, the concentration index for the three diseases based on an employment status of population is indicated. The results indicate that respiratory and circulatory diseases show no statistically significant association with employment status. However, for neoplasms, the results are statistically significant and negative, indicating higher prevalence of neoplasms in populations living in areas with higher unemployment levels (Table 3). Additionally, the concentration curve for neoplasms lies above the line of equality (Figure 1).

##### Disease Proportion Based on Apartment Ownership

**Figure 2:**
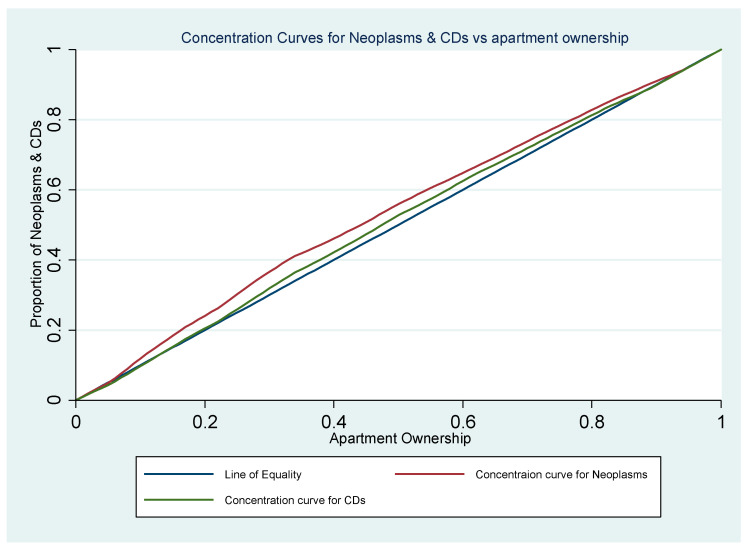
The concentration curve based on proportion of patients treated with neoplasms and circulatory diseases and the apartment ownership in Pécs City.

Table 4 summarizes the concentration index for respiratory diseases, neoplasms, and circulatory diseases in relation to apartment ownership within the population. While the results for respiratory diseases are not statistically significant, both neoplasms and circulatory diseases show significant association with apartment ownership. The negative concentration index indicates that neoplasms and circulatory diseases are more prevalent in areas with fewer apartment owners. Additionally, the concentration curves for both neoplasms and circulatory diseases lie above the line of equality (Figure 2).

## 4. Discussion

This study examined hospitalization data to assess the presence of health inequity in the distribution of respiratory system diseases, circulatory diseases, and neoplasms among the adult population living in different areas of Pécs, Hungary.

This study revealed that based on employment status, neoplasms are more frequent among the population living in zip codes where many are unemployed than their counterparts. This is in line with the finding of the study carried out throughout 17 European countries from 1980–2015, where disadvantaged individuals systematically suffer from significantly higher mortality rates and lower survival rates than groups with higher socioeconomic status for the vast majority of cancers [21]. Similarly, findings from the European Social Survey (2014) hav3 indicated that at the pooled European level, a social gradient in health was observed for cancer and other non-communicable diseases [22].

Consistent with our results, a study carried out in the United States showed that individuals in more deprived areas or lower socioeconomic status faced higher cancer mortality and incidence rates than their more affluent counterparts [23]. This could be attributed to the fact that a low socioeconomic level is associated with risk factors for cancers, as shown in the European study; accordingly, a socioeconomic gradient can be observed for most risk factors, with people with low socioeconomic status being more likely to use tobacco, be overweight, have an unhealthy diet, and be physically inactive than people with higher socioeconomic status in EU+2 countries [24].

Additionally, our study revealed that neoplasms and circulatory diseases are more prevalent in areas with fewer apartment owners. This finding is consistent with the finding of a Germany study that described financial instability to be associated with a higher incidence of circulatory diseases [25]. Similarly, a multi-center study across Europe revealed compelling evidence of a widening gap in circulatory disease and other chronic disease outcomes between advantaged and disadvantaged subgroups across Europe [26]. Similarly, a study conducted in the United States found that low education, low income, and unemployment at both individual and area levels were associated with an increased risk of developing circulatory diseases, particularly atherosclerotic cardiovascular disease [27]. Persistent inequality in mortality from circulatory disease is likely to be influenced by the higher rates of smoking, unhealthy dietary habits, alcohol consumption, physical inactivity, and obesity in socially disadvantaged subgroups, along with a complex interplay of genetics, childhood environment, material living conditions, social and psychological factors, and access to health care [28,29].

It is known that morbidity and mortality risks are higher among the unemployed compared to the employed population [30]. The links between housing and health are now known to be strong and multifaceted, and to generally span across four key pillars: stability, affordability, quality and safety, and neighborhood opportunity [31].

Health and health inequity are the result of more than individual, interpersonal, or biological factors. Social, economic, environmental, and policy drivers also determine the health status of individuals and populations [32]. Based on this finding and further studies, policy makers should build multisector stakeholder engagement, extend beyond individual outcomes to community and system level outcomes, and expand methods for implementing, evaluating, and disseminating multilayered, multifaceted interventions. The tailored interventions such as targeted healthcare funding and employment programs for the unemployed and National Housing Trust Fund for those who do not own houses require collaboration among healthcare providers, employers, governors, and policymakers.

## 5. Limitations of the Study

Firstly, this study is based on secondary data, and data on variables like behavioral and environmental factors are not recorded. This could lead to confounding bias and the results should be interpreted cautiously. Secondly, the results indicate correlation, which does not imply causation.

## 6. Conclusions

This study identified significant health inequity in the distribution of neoplasms and circulatory diseases among the adult population of Pécs, especially in areas where the low socioeconomic segment of the population lives. To reduce inequity in the city, efforts should be made to establish tailored interventions such as targeted healthcare funding and employment programs for the unemployed, and National Housing Trust Fund for those who do not own houses through multisectoral collaboration among healthcare providers, employers, governors, and policymakers.

## Figures and Tables

**Table 1:** The difference and ratio of proportion of patients treated with selected diseases by zip codes grouped by socio-demographic variables at Pécs City, 2019.

Disease	Zip Codes Grouped by Education					Difference (Cat 5 − Cat 1)	Ratio (Cat 5/Cat 1)
	Cat 1	Cat 2	Cat 3	Cat 4	Cat 5		
Respiratory disease	0.2288	0.2622	0.2451	0.2515	0.2378	0.009	1.0393
Circulatory disease	0.3372	0.3551	0.3358	0.3581	0.2922	−0.045	0.8665
Neoplasms	0.0202	0.0247	0.0263	0.0281	0.0204	0.0002	1.0099
	**Zip codes grouped by Employment**						
	**Cat 1**	**Cat 2**	**Cat 3**	**Cat 4**	**Cat 5**		
Respiratory disease	0.2479	0.2568	0.2406	0.2500	0.2617	0.0138	1.0557
Circulatory disease	0.3426	0.3676	0.3375	0.3362	0.3165	−0.0261	0.9239
Neoplasms	0.0275	0.0268	0.0234	0.0230	0.0202	−0.0073	0.7352
	**Zip codes grouped by apartment ownership**						
	**Cat 1**	**Cat 2**	**Cat 3**	**Cat 4**	**Cat 5**		
Respiratory disease	0.2549	0.2479	0.2481	0.2712	0.2406	−0.0143	0.9440
Circulatory disease	0.3496	0.3732	0.3607	0.3360	0.3186	−0.0310	0.9113
Neoplasms	0.0300	0.0295	0.0243	0.0215	0.0215	−0.0085	0.7163

Cat 1 indicates areas with the lowest proportion of educated residents, employed residents, or those with owner-occupied apartment.

**Table 2:** Concentration index based on education.

Disease	Concentration Index	95% Confidence Interval Lower Bound	95% Confidence Interval Upper Bound	*p*-Value
Respiratory diseases	0.0020	−0.0199	0.0239	0.8610
Neoplasms	0.0254	−0.0283	0.0791	0.3683
Circulatory diseases	−0.0130	−0.0421	0.0161	0.3955

**Table 3:** Concentration index based on employment status.

Disease	Concentration Index	95% Confidence Interval Lower Bound	95% Confidence Interval Upper Bound	*p*-Value
Respiratory diseases	0.0029	−0.0183	0.0242	0.7914
Neoplasms	−0.0528	−0.0975	−0.0080	0.0345 *
Circulatory diseases	−0.0148	−0.0418	0.0123	0.3001

* = significant result at *p* < 0.05 level.

**Table 4:** Concentration index based on apartment ownership.

Disease	Concentration Index	95% Confidence Interval Lower Bound	95% Confidence Interval Upper Bound	*p*-Value
Respiratory diseases	−0.0098	−0.0303	0.0108	0.3654
Neoplasms	−0.0742	−0.1102	−0.0382	0.0010 *
Circulatory diseases	−0.0280	−0.0520	−0.00389	0.0369 *

* = significant result at *p* < 0.05 level.

## Data Availability

The datasets used and/or analyzed during the current study are available from the corresponding author upon reasonable request.

## Supplementary Materials

The following supporting information can be downloaded at: https://www.mdpi.com/article/10.3390/healthcare13060681/s1, Table S1: Socio-demographic characteristics of the population in each zip code of Pécs City, 2019. Figure S1. The bar chart showing the proportion of patients treated for neoplasms in Pécs City, 2019. Figure S2. The bar chart showing the proportion of patients treated for circulatory diseases in Pécs City, 2019. Figure S3. The bar chart showing the proportion of patients treated for respiratory system diseases in Pécs City, 2019.
