# Supplementary material for: Health Inequity in the Distribution of Diseases Among Adults in the City of Pécs, Hungary, 2024
Source: Healthcare (Basel). 2025 Mar 20;13(6):681. doi: 10.3390/healthcare13060681 (PMC11942578; doi:10.3390/healthcare13060681)
Supplement: Supplementary file 1 [file healthcare-13-00681-s001.zip › healthcare-3492285-supplementary.pdf]

Supplementary Materials

Table S1: Socio-demographic characteristics of the population in each zip code of Pécs City, 2019.

Zip code	Variables				Total population
	Education: University and College completed (%)	Education: Grade 8 and below (%)	Employed (%)	Ownership of apartment (%)	
**7621**	1682 (30.66)	1758 (32.05)	1773 (32.32)	3390 (61.79)	5486
**7622**	603 (21.09)	724 (25.33)	875 (30.62)	1781 (62.32)	2858
**7623**	2130 (27.70)	2117 (27.53)	2874 (37.37)	6141 (79.86)	7690
**7624**	3429 (28.44)	2890 (23.97)	3918 (32.49)	7858 (65.17)	12058
**7625**	2179 (34.13)	1904 (29.82)	2511 (39.33)	5616 (87.96)	6385
**7626**	953 (21.36)	1601 (35.88)	1720 (38.56)	3158 (70.79)	4461
**7627**	1893 (21.19)	3366 (37.68)	3556 (39.81)	8106 (90.75)	8932
**7628**	675 (11.00)	2875 (46.85)	2041 (33.26)	4660 (75.95)	6136
**7629**	807 (11.45)	3270 (46.42)	2448 (34.75)	5417 (76.89)	7045
**7630**	928 (13.66)	2812 (41.38)	2638 (38.82)	6233 (91.73)	6795
**7631**	611 (15.35)	1432 (35.98)	1703 (42.79)	3812 (95.78)	3980
**7632**	5478 (15.32)	12086 (33.79)	14964 (41.84)	29299 (81.92)	35767
**7633**	3265 (19.02)	5460 (31.81)	5981 (34.85)	12275 (71.52)	17163
**7634**	2317 (24.27)	3115 (32.63)	3983 (41.72)	8957 (93.83)	9546
**7635**	2060 (33.66)	1894 (30.95)	2651 (43.32)	5904 (96.47)	6120
**7636**	1595 (18.04)	2780 (31.45)	4087 (46.23)	7713 (87.25)	8840
**7691**	320 (7.20)	2019 (45.45)	1639 (36.90)	4041 (90.97)	4442
**7693**	-	730 (43.48)	590 (35.14)	1582 (94.22)	1679

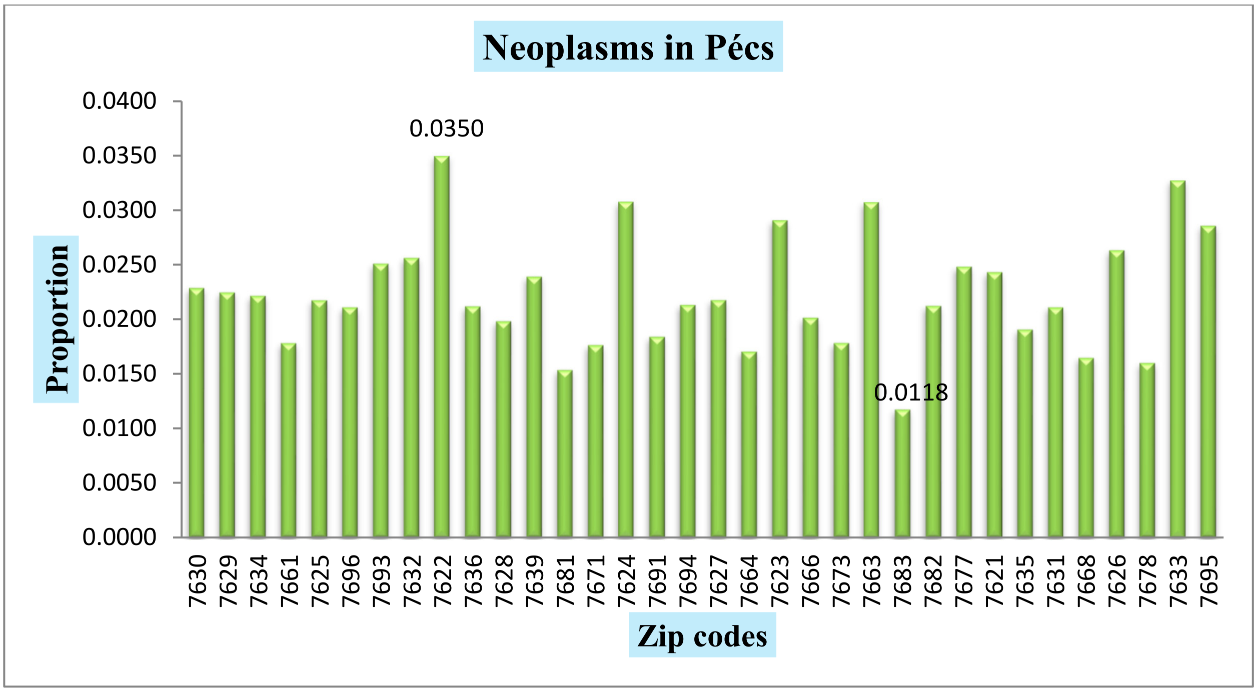

Figure S1. The bar chart showing the proportion of patients treated for neoplasms in Pécs City, 2019.

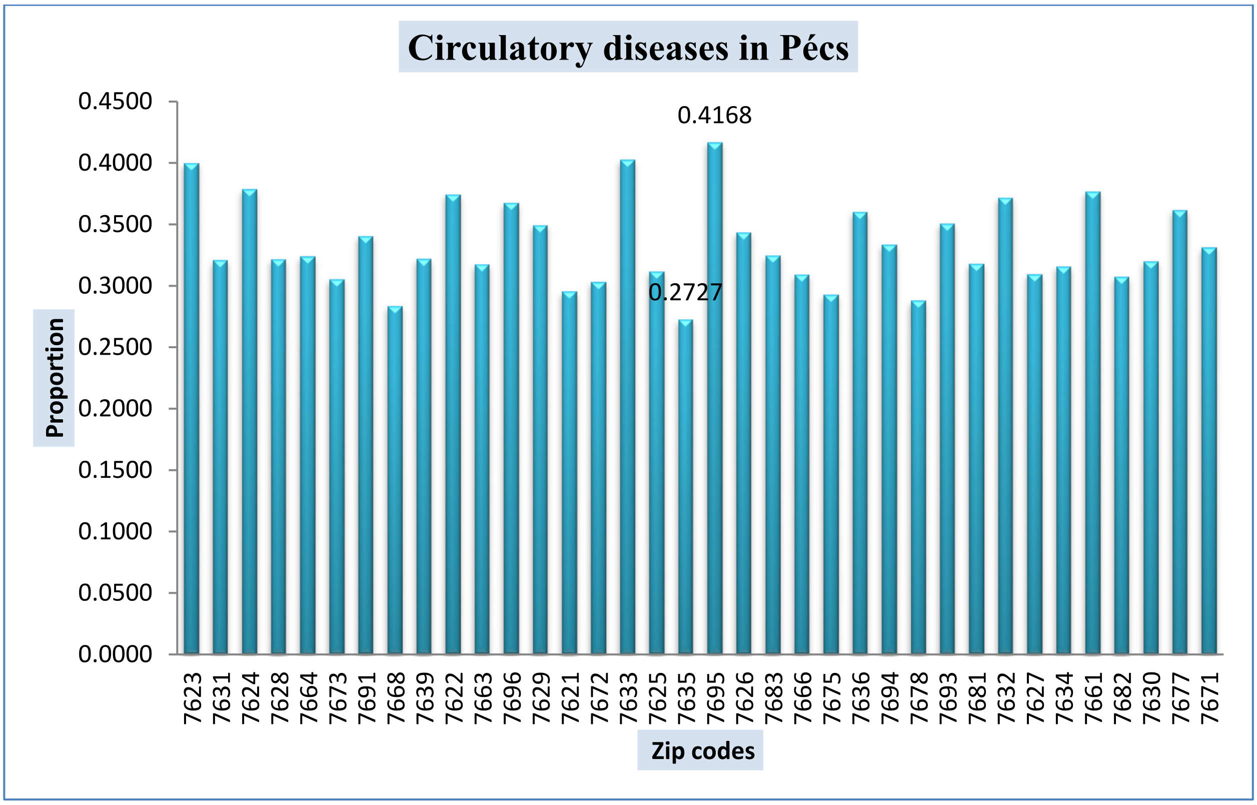

Figure S2. The bar chart showing the proportion of patients treated for circulatory diseases in Pécs City, 2019.

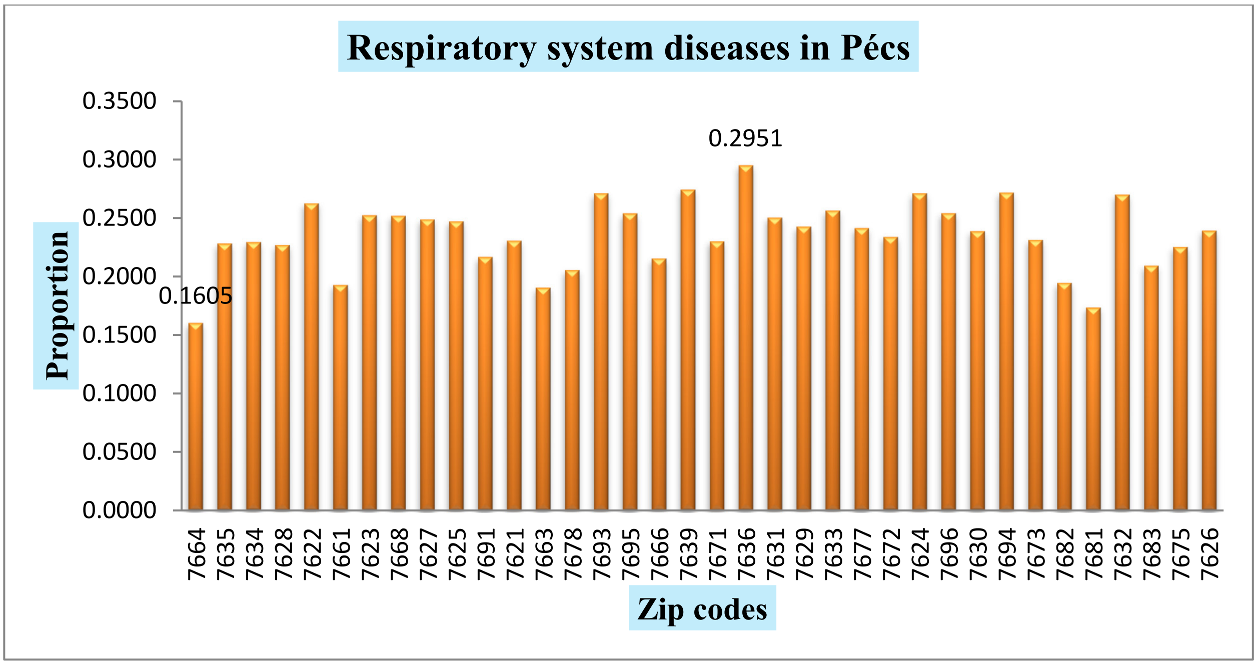

Figure S3. The bar chart showing the proportion of patients treated for respiratory system diseases in Pécs City, 2019.
